# In Vitro and In Vivo Neurotoxicity of Prion Protein Oligomers

**DOI:** 10.1371/journal.ppat.0030125

**Published:** 2007-08-31

**Authors:** Steve Simoneau, Human Rezaei, Nicole Salès, Gunnar Kaiser-Schulz, Maxime Lefebvre-Roque, Catherine Vidal, Jean-Guy Fournier, Julien Comte, Franziska Wopfner, Jeanne Grosclaude, Hermann Schätzl, Corinne Ida Lasmézas

**Affiliations:** 1 Commissariat à l'Energie Atomique, Fontenay-aux-Roses, France; 2 Institut National de la Recherche Agronomique, Jouy-en-Josas, France; 3 Department of Infectology, The Scripps Research Institute, Jupiter, Florida, United States of America; 4 Institute of Virology, Technical University of Munich, Munich, Germany; 5 Institut Pasteur, Paris, France; Roslin Institute, United Kingdom

## Abstract

The mechanisms underlying prion-linked neurodegeneration remain to be elucidated, despite several recent advances in this field. Herein, we show that soluble, low molecular weight oligomers of the full-length prion protein (PrP), which possess characteristics of PrP to PrPsc conversion intermediates such as partial protease resistance, are neurotoxic in vitro on primary cultures of neurons and in vivo after subcortical stereotaxic injection. Monomeric PrP was not toxic. Insoluble, fibrillar forms of PrP exhibited no toxicity in vitro and were less toxic than their oligomeric counterparts in vivo. The toxicity was independent of PrP expression in the neurons both in vitro and in vivo for the PrP oligomers and in vivo for the PrP fibrils. Rescue experiments with antibodies showed that the exposure of the hydrophobic stretch of PrP at the oligomeric surface was necessary for toxicity. This study identifies toxic PrP species in vivo. It shows that PrP-induced neurodegeneration shares common mechanisms with other brain amyloidoses like Alzheimer disease and opens new avenues for neuroprotective intervention strategies of prion diseases targeting PrP oligomers.

## Author Summary

Prion diseases are transmissible neurodegenerative diseases caused by an infectious agent thought to be composed mainly of a host protein, the prion protein (PrP). The mechanisms of neurodegeneration prevailing in these diseases are not well understood. In the present study, we demonstrate that small PrP aggregates, called oligomers, cause the death of neurons in culture and after injection in vivo. On the contrary, larger PrP aggregates, visualized as fibrils by electron microscopy, do not cause the death of cultured neurons and are much less toxic than PrP oligomers in vivo. We propose that the PrP oligomers exert their toxicity by disturbing neuronal membranes, as well as by an excessive intracellular concentration leading to the generation of death signals (also called apoptotic signals) by the cell. Moreover, the use of antibodies recognizing a certain portion of the PrP oligomers could prevent neuronal death. This study assigns prion diseases to the same group of diseases as Alzheimer disease, in which protein oligomers constitute the major trigger of the neurodegenerative process, and suggests new possible neuroprotective approaches for therapeutic strategies.

## Introduction

Transmissible spongiform encephalopathies are infectious neurodegenerative diseases. They are characterized by the accumulation in the brain, and sometimes the lymphoid tissues [1,2], of an abnormally structured form (PrPsc) of the host prion protein (PrP) [3]. PrPsc may constitute the infectious agent, also called prion, entirely [4] or in part [5,6]. The mechanism of neurodegeneration that ultimately leads to neuronal death and the occurrence of clinical symptoms, however, is still not known [7,8]. It has become apparent that immunohistochemically detectable PrPsc aggregates, of various sizes ranging from fine granular deposition to amyloid plaques, do not represent the neurotoxic entity of prion diseases. Indeed, PrPsc is not detectable in some cases of fatal familial insomnia [9], in lethal scrapie-like disease in mice overexpressing mutant PrP transgenes [10], in wild-type mice inoculated with bovine spongiform encephalopathy [11,12] or fatal familial insomnia [13], and in prion-infected mice with a P101L mutation in their PrP gene [14]. The hypothesis has been made earlier that the critical events in pathogenesis occur at the submicroscopic level [15].

On the other hand, PrP peptides comprising the hydrophobic domain (residues 106–126) of PrP are toxic to cultured neurons [16–19]. N-terminally truncated PrP also triggers neuronal death in the absence of expression of the normal form of the protein [20]. This shows that PrP has intrinsic properties that could render the protein toxic under certain conditions.

There is growing evidence that in other brain amyloidoses, prefibrillar soluble protein aggregates, rather than insoluble fibrils, are toxic [21–24]. In vivo, 56-kD dodecameric assemblies of Aß1–42, dubbed Aß* (of note, PrP* has also been proposed as the biologically reactive form of PrP [25]), have been shown to be associated with memory deficits in a murine model of Alzheimer disease and to cause transient memory impairment after injection in the brains of rats [26]. In a zebrafish model, expression of polyQ-expanded fragments of huntingtin lead to their accumulation as large SDS-insoluble cell inclusions; however, apoptotic cells are devoid of visible aggregates. Remarkably, the treatment with two anti-prion compounds prevented the formation of insoluble aggregates but did not suppress abnormal embryo morphology or cell death, strongly suggesting that upstream soluble huntingtin assemblies constitute the toxic culprit [27].

Recently, soluble oligomers presenting an enriched ß-sheeted structure were proposed as intermediates in the amyloidogenesis process featured in prion diseases [28–31]. PrP oligomers were toxic in vitro [32,33].

We wanted to further investigate the hypothesis that prion diseases share a common mechanism of neurodegeneration with other brain amyloidosis, and set out to study the toxicity of PrP oligomers in vitro and in vivo in the presence or absence of endogenous PrP expression. We found that PrP oligomers exhibit considerably higher toxicity than PrP fibrils both in vitro and in vivo. PrP monomers were nontoxic. The toxicity occurred whether or not the neurons expressed PrPc. The toxicity of PrP oligomers could be abrogated by blocking the hydrophobic domain at the surface of the oligomers. We propose a comprehensive model of the possible mechanisms of prion-induced neurodegeneration.

## Results

### Production and Characterization of PrP Oligomers

Two types of PrP oligomers were produced by either thermal refolding or expression of PrP in form of a tandem repeat.

**Figure 1:**
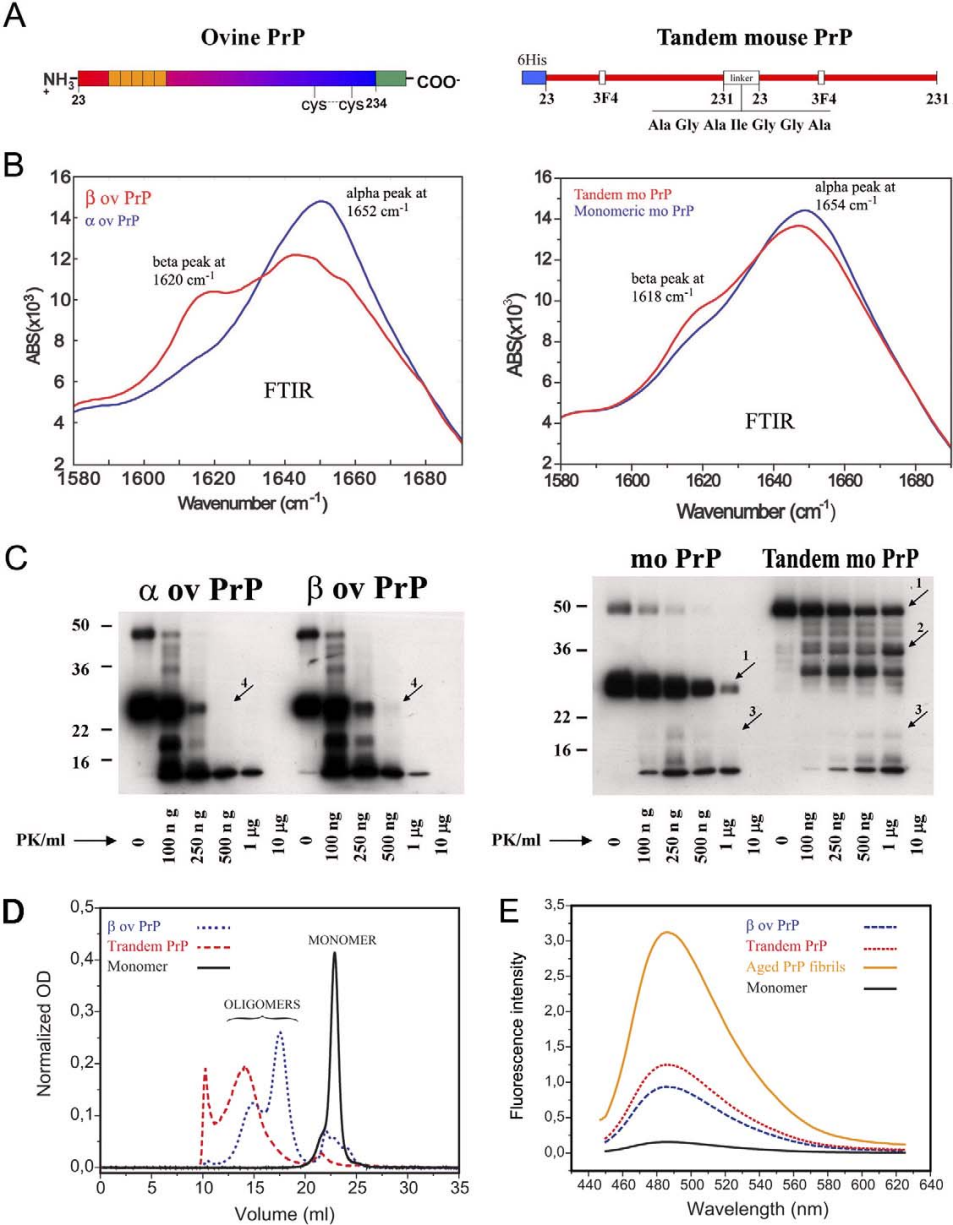
Characterization of PrP Species (A) Molecular representation of the PrP proteins used. Left: ovine PrP. Right: covalently linked tandem PrP constructed by associating two mature murine PrP sequences (aa 23–231, not containing N- and C-terminal signal peptides) with a 7–amino acid linker sequence in a head-to-tail manner. The proteins were purified as described in the Materials and Methods section. (B) Fourier transform infrared spectra (FTIR) of recombinant PrP proteins. Left: ovine α-PrP (blue) and ß-PrP (red). Right: monomeric (blue) and tandem (red) mouse PrP proteins. The results confirm the ß-sheeted conformation of the ß-PrP and indicate that the tandem PrP adopts a ß-sheet-enriched conformation in physiological buffers. (C) PK sensitivity of PrP species. PrP proteins were subjected to a PK digestion of 15 min at various concentrations of PK ranging from 100 ng/ml to 10 μg/ml. Both ovine ß-PrP (left) and mouse tandem PrP (right) exhibited partial protease resistance. The results are representative of at least two independent experiments. The numbered arrows point to locations where differences in PK resistance can be observed between monomeric and oligomeric PrP. D) Size exclusion chromatography performed on ovine PrP (black and blue lines) and covalently linked tandem PrP (red line) in PBS buffer, immediately after purification. The black line corresponds to the α-helical ovine PrP monomers; the blue line corresponds to the refolded, ß-sheeted ovine PrP preparations (very small monomeric peak, major bimodal oligomeric peak with an excess of 12 mers compared to the 36 mers); the red line corresponds to the murine tandem PrP preparations (very small monomeric peak, large oligomeric peak around the 36-mer peak of the ovine PrP oligomers, and sharp peak on the left corresponding to highly aggregated molecules that eluted in the column void volume,V0). (E) Thioflavine assay, which measures the fluorescence intensity of thioflavine bound to amyloiditic protein. Orange, fibrils of ovine PrP; blue, ovine PrP oligomers 15 μM; red, murine tandem PrP oligomers 15 μM; black, ovine monomers 15 μM.

A recombinant ovine PrP (23–234) was generated and converted into a ß-sheeted form (ß-PrP) (Figure 1A and 1B, left panel) by thermal refolding (see Materials and Methods and [34]). Both the ß-sheeted and the α-helical conformers had mobilities corresponding to a molecular weight of about 25 kDa by SDS-PAGE (Figure 1C, left panel).

Another type of PrP oligomer was created by exploiting the finding that dimerization has been described as a primary event in the PrP conversion and aggregation process [35]. Two monomeric PrP units were covalently linked head to tail via a flexible linker (Figure 1A, right panel). This protein displayed a molecular weight double that of the monomeric PrP in the presence of detergents (Figure 1C, right panel). The tandem murine PrP presents a higher ß-sheeted content than the monomeric PrP as judged by the increase in the 1,618 cm^−1^-peak (Figure 1B, right panel). Size exclusion chromatography of both types of PrP preparations in physiological buffers showed that they were exclusively in an oligomeric state ([36] and Figure 1D). In summary, the tandem mouse PrP oligomerized spontaneously after purification, while the ovine PrP oligomerized after heating. These results are consistent with the recent finding that α-PrP molecules convert into ß-sheeted oligomers with a molten globule intermediate in the absence of any detectable ß-sheeted monomeric PrP intermediate [37]. Both types of PrP oligomers showed slightly enhanced thioflavine T binding (Figure 1E), strongly suggesting that they were on the pathway of amyloid fibril formation (see below).

### Oligomeric PrP Is Slightly Proteinase K Resistant

We investigated whether oligomeric PrP was more resistant to proteinase K (PK) digestion than monomeric PrP. Each type of PrP was submitted to a range of PK concentrations. Both murine and ovine PrP oligomers exhibited partial resistance to PK digestion when compared to their respective monomeric counterparts (Figure 1C), as shown by i) the difference in the degradation profile between the tandem PrP oligomers and the monomer at 500 ng/ml and 1 μg/ml (the degradation of the tandem levels out, whereas the monomer continues to be degraded (compare arrows #1); ii) a PK-resistant core of the tandem of 36 kDa, which appears with increasing PK concentrations (see arrow #2); iii) the 20-kDa degradation product from the tandem increases with increasing concentrations of PK contrarily to the same 20-kDa product from the monomer (compare arrows #3); and iv) the residual ∼25-kDa band of the ovine ß-PrP observed at the PK concentration of 500 ng/ml (albeit weak, this signal was reproducible between experiments), contrasting with the complete digestion of ovine PrP monomers at the same concentration (arrows #4).

### Oligomeric PrP Is Neurotoxic to Mouse Cortical Neurons

**Figure 2:**
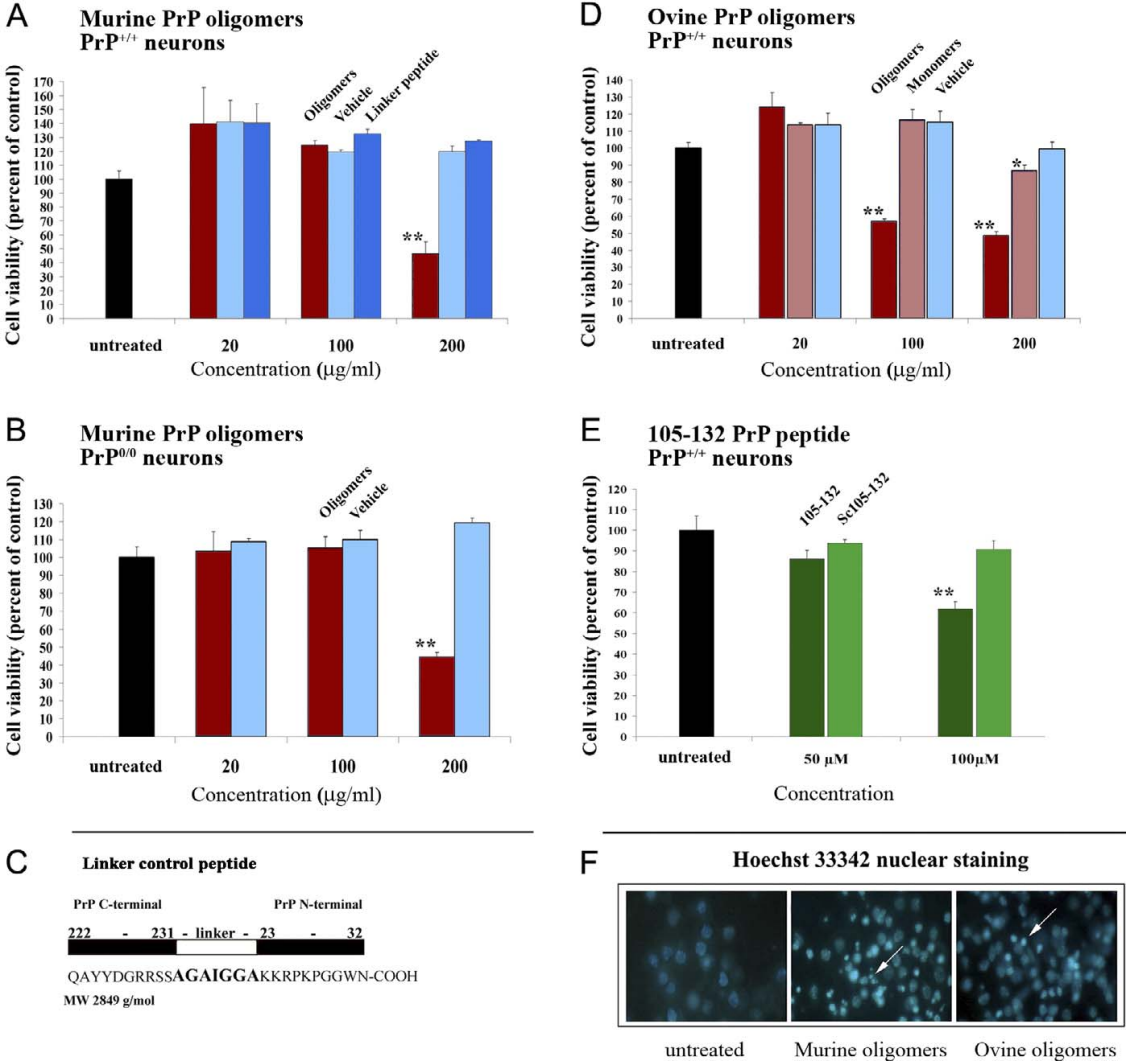
Neurotoxicity of Oligomeric PrP (A–C) E15 cortical neurons from PrP^+/+^ (A) or PrP^0/0^ (B) were exposed to various concentrations of murine PrP oligomers (red bars) for 72 h, and then cell viability was measured using MTT. Untreated neurons (black bars) and neurons treated with either equivalent volumes of vehicle solution (light blue bars) or a peptide mimicking the linker region of the mouse tandem PrP (dark blue bars) were chosen as controls (see [C] for molecular details of the peptide). (D) Cell viability measurements of neurons treated with either ovine PrP oligomers (dark red bars) or ovine PrP monomers (light red bars). The black and light blue bars correspond to untreated and treated with vehicle solution controls, respectively. (E) To compare the toxicity of the PrP preparations with a known toxic PrP peptide, PrP^+/+^ neurons were incubated with the PrP peptide 105–132 (dark green bars) and its scrambled version (light green bars) at two concentrations reported to induce neuronal death [18]. The cell viability results are expressed as a percentage relative to the untreated control (black bar) + SEM. The results are representative of at least two independent experiments performed with triplicate samples. The significance of the results was evaluated using a two-tailed unpaired student *t*-test with Welch corrections when needed (significant = *, 0.01 < *p* ≤ 0.05; very significant = **, *p* ≤ 0.01). (F) Hoechst 33342 nuclear staining of embryonic cortical neurons incubated with toxic recombinant proteins. Left: No protein control, showing normal fully rounded cell nuclei. Middle: incubated with mouse PrP oligomers. Right: incubated with ovine PrP oligomers. Condensed nuclei characteristic of apoptotic cells are seen in treated cells (white arrows).

We first analyzed the neurotoxic properties of the PrP oligomers in a murine primary cortical neuron model. Exposure of the neurons to both types of PrP oligomers resulted in a loss of nearly 50% of the neurons when compared to the untreated control cells, at a dose of 200 μg/ml (Figure 2A) and 100 μg/ml (Figure 2D), both corresponding to a concentration of 3 μM. Hence, oligomeric PrP exhibited a roughly 30-fold higher toxic effect than the PrP peptide 105–132, which inserts into cellular membranes, and which induced 40% neuronal death at 100 μM (Figure 2E). The same levels of toxicity were observed in PrP^0/0^ neurons, indicating that the effect was not dependent on the expression of PrP by the cells (Figure 2B for murine PrP oligomers, data not shown for ovine PrP oligomers). We then wanted to verify that the seven–amino acid linker or the His expression tag present in the murine PrP oligomers were not responsible for their toxicity. Neither the linker, flanked on both sides by ten residues from the N- and C-terminal regions of the PrP sequence, (Figure 2C) nor grb-2 [38], an irrelevant protein of the same size as PrP (217 aa), generated in the same expression system as the tandem PrP and carrying the same His-tag, were toxic to the neurons (Figure 2A and data not shown).

To understand the type of neuronal death induced by these oligomers, the cells were analyzed morphologically using nuclear staining. Neuronal cultures treated with murine or ovine PrP oligomers (Figure 2F) revealed a high quantity of cells exhibiting condensed and fragmented chromatin, a hallmark of apoptosis.

### The Hydrophobic Domain of PrP Oligomers Is Essential for Toxicity

**Figure 3:**
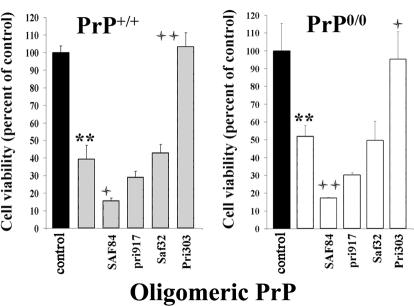
Rescuing of Oligomeric PrP-Induced Toxicity by Domain-Specific PrP Antibodies Embryonic cortical neurons from PrP^+/+^ (left, light gray bars) or PrP^0/0^ (right, white bars) were incubated with 200 μg/ml (3 μM) of mouse PrP oligomers, in the presence or absence of PrP-specific monoclonal antibodies directed against different regions of the PrP protein (SAF32: 59–92; Pri303: 106–126; SAF84: 161–170; Pri917: 217–221). The results are expressed in terms of percentage relative to the untreated control (black bar). The graphs are representative of at least two independent experiments performed with triplicate samples. The significance of the results was evaluated using a two-tailed unpaired student *t*-test with Welch corrections when needed. * indicates significance of values in relation to the untreated control (significant = *, 0.01 < *p* ≤ 0.05; very significant = **, *p* ≤ 0.01). + indicates significance of values in relation to the toxic dose (significant = , 0.01 < *p* ≤ 0.05; very significant = ++, *p* ≤ 0.01).

To identify the domains of PrP oligomers accountable for the observed toxicity, we occluded different regions of PrP oligomers by incubating them with a series of domain-specific PrP antibodies. Neurons exposed to murine or ovine PrP oligomers were fully protected against cell death using the monoclonal antibody Pri303 directed against the domain 106–126 of PrP (Figure 3 and data not shown). In contrast, incubation with the PrP monoclonal antibodies SAF84 (aa 161–170), Pri917 (aa 217–221), or SAF32 (aa 59–92) had no protective effect. These data show that the exposure of hydrophobic domain of PrP at the surface of the PrP oligomers is required for the neurotoxic mechanism. The effect of the various antibodies was similar on neurons expressing PrP or not (Figure 3), confirming in an independent experiment that PrP oligomers are toxic to primary neurons regardless of the expression of PrPc, and showing that the prevailing neurotoxic mechanism can not be counteracted by endogenous PrPc present at the cell surface.

### PrP Fibrils Are Not Toxic on Primary Cortical Neurons

**Figure 4:**
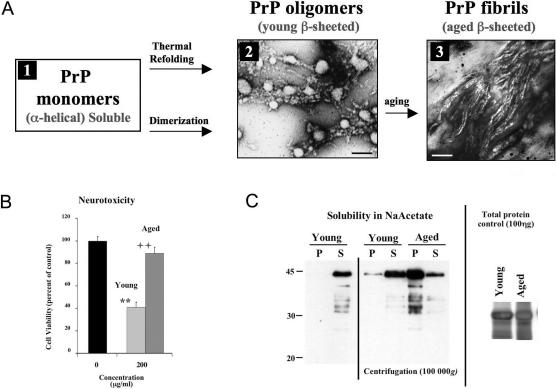
Relationship between PrP Ultrastructure, Solubility, and Neurotoxicity (A) Electron microscopy analyses showing that PrP oligomers form granular aggregates and protofibrils (panel 2, scale bar = 100 nm). Upon aging, they assemble into long robust fibrils of PrP (panel 3, scale bar = 50 nm). Ovine PrP oligomers are shown in the pictures (panels 2 and 3), but similar results were obtained with murine tandem PrP oligomers. (B) WST-1 cell viability measurements of cortical neurons treated with young (oligomeric) or aged (fibrillar) PrP showing that protofibrillar/oligomeric PrP is highly toxic, whereas fibrillar PrP is not toxic. The significance of the values was evaluated using a two-tailed unpaired student *t*-test with Welch corrections when needed. * indicates significance of values in relation to the untreated control (very significant = **, *p* ≤ 0.01). + indicates significance of values when compared with the toxic dose (very significant = ++, *p* ≤ 0.01). (C) Solubility properties of PrP oligomers (young) and PrP fibrils (aged) in sodium acetate. Aged PrP forms insoluble aggregates as seen from the pellet fraction (P = pellet, S = supernatant). A total protein control shows that equal amounts of each protein were used (right panel). Murine PrP oligomers are shown in (B and C).

We then wanted to determine whether toxicity was limited to oligomeric PrP species or if PrP fibrils could also be toxic. We established that aging of PrP oligomers (a temperature-dependent process, taking from minutes at 50 °C to one month at 4 °C) led to their polymerization into PrP fibrils. Figure 4A shows by electron microscopy that PrP oligomers exhibit a mixed granular and protofibrillar structure, while aged preparations form long mature fibrils 15 nm in diameter. These fibrils had amyloid properties as they strongly bound to thioflavine T (Figure 1E); they also showed enhanced PK resistance when compared to the PrP oligomers (not shown). Toxic PrP oligomers were soluble in sodium acetate, whereas aged PrP proteins were insoluble (Figure 4C, compare young and aged in the pellet fraction-P-). Fibrillar PrP preparations were not toxic, in contrast with oligomeric PrP (Figure 4B).

### PrP Oligomers Exhibit Higher Neurotoxicity than PrP Fibrils In Vivo, and the Toxicity Is Independent of Endogenous PrP Expression

**Figure 5:**
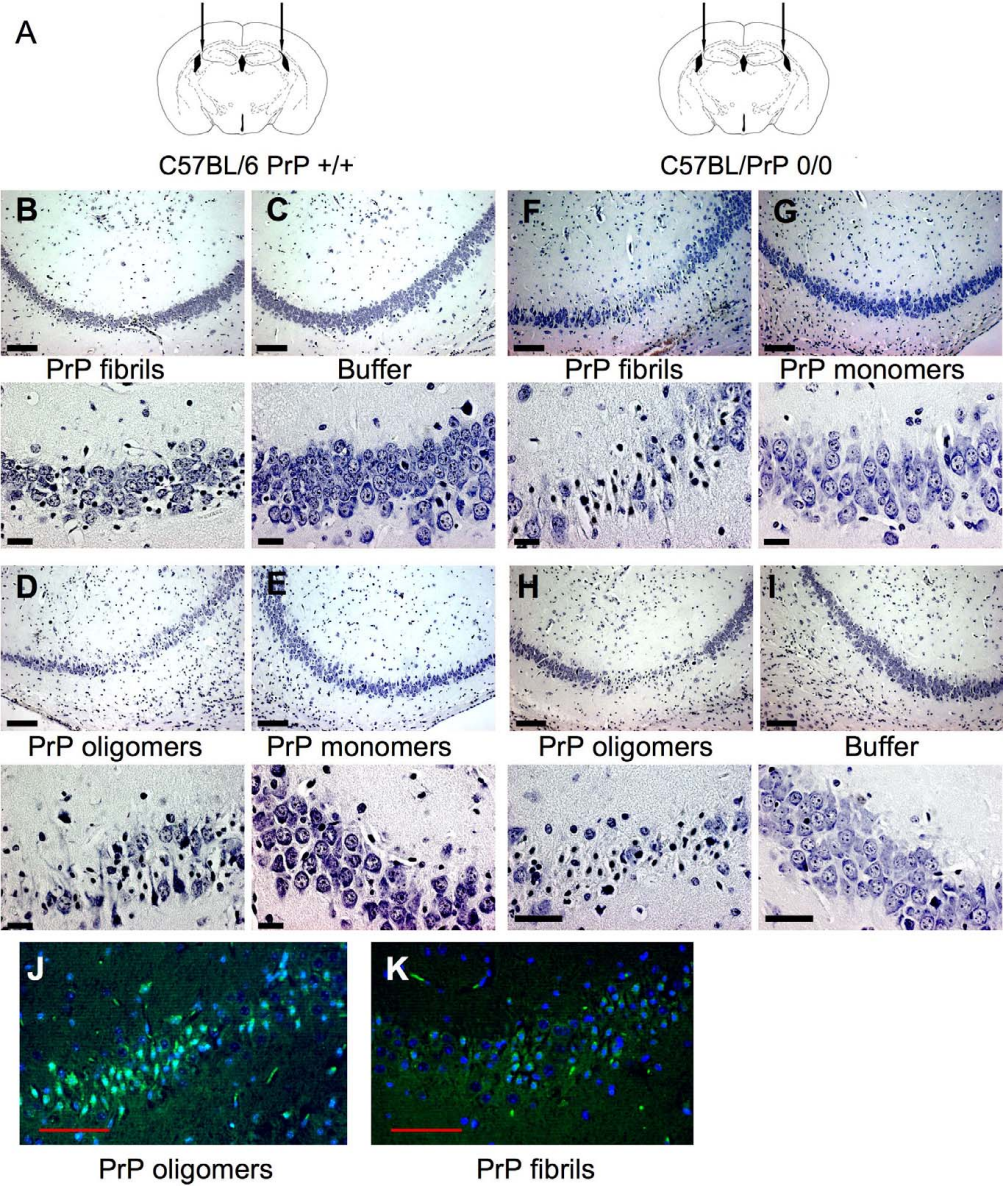
In Vivo Neurotoxicity of Ovine PrP Oligomers and Fibrils (A) Schematic representation of a coronal section of a mouse brain with arrows indicating the injection sites of the PrP preparations. (B–I) Nissl-like staining (gallocyanine) of the hippocampal region CA_2_-CA_3_ from the brains of mice injected with the PrP preparations or control buffer. Top panels represent the low magnification image (scale bar = 100 μm) and bottom panels the high magnification image (scale bar = 20 μm) of the lesioned regions (and anatomically corresponding region for the buffer and nontoxic PrP monomers). (B–E) Wild-type C57BL/6 mice, (F–I) PrP0/0 C57BL/6 mice. (J and K) Higher toxicity of PrP oligomers versus PrP fibrils (wild-type C57BL/6 mice shown). Apoptotic pyramidal neurons in the lesioned hippocampal region have been labeled with the ApopTag BrdU kit and appear in green (scale bar = 50 μm).

**Figure 6:**
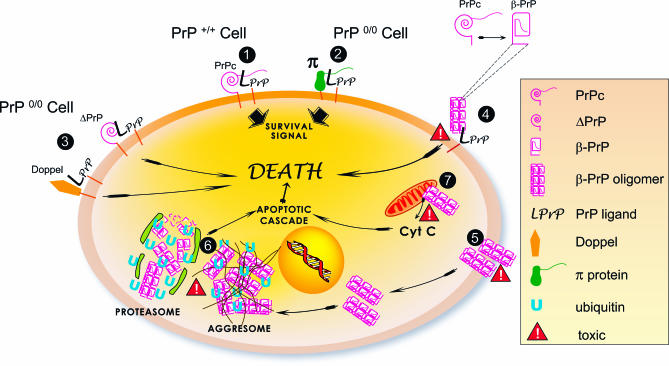
Model for the Mechanism of Oligomeric PrP-Induced Neurodegeneration The scheme is derived from previous findings by other authors and has been complemented with mechanisms suggested by our data. (1) PrP interacts with its ligand LPrP on the cell surface to generate a signal necessary for cell survival. PrP binds to its natural ligand LPrP via its globular domain, while the interaction of the flexible N-terminus of PrP with LPrP triggers signal transduction. (2) In a PrP knock-out cell, π can replace PrP for both binding and signal transduction [20,64]. (3) In experimental models where truncated forms of PrP (ΔPrP) or Doppel (Dpl, a member of the PrP supergene family harboring partial sequence and structure similarity with PrP) are expressed on a PrP^0/0^ background, binding of ΔPrP or Dpl to LPrP occurs, but not signal transduction in the absence of a complete N-terminus [20,56,57,65]. Suppression of PrP or π signaling triggers cell death. (4) PrP oligomers present an altered interaction with LPrP. They bind, but do not trigger signal transduction, and thus by competition prevent PrPc (PrP^+/+^ cells) or π (PrP^0/0^ cells) binding to LPrP, resulting in cell lethality. (5) The hydrophobic domain of PrP at the surface of the oligomers enhances their insertion in the cellular membrane. This leads to membrane dysfunction, and hypothetically to the formation of pore-like structures [19,54] inducing toxic signals. (6) At high intracellular concentration, PrP oligomers accumulate in the aggresome [61] and saturate the proteasomal or other degradation pathways of the cell, leading to the generation of apoptotic signals. (7) The hydrophobic domain of PrP oligomers interact abnormally with mitochondrial membranes, leading to the release of cytochrome C (Cyt C) triggering the apoptotic cascade [66].

To investigate the toxicity of different forms of PrP in vivo, stereotaxic subcortical injections of oligomeric or fibrillar PrP were carried out in the right hemispheres (ipsilateral) of C57BL/6 PrP^+/+^ or C57BL/6 PrP^0/0^ mice, and monomeric PrP or buffer alone was injected in the left hemispheres (contralateral). Table 1 and Figure 5 describe the experiments performed with ovine PrP preparations. The injection scheme is summarized in Table 1. Figure 5A shows the precise site of injection, just above the CA2 region of the hippocampus. The effect of the PrP preparations on neuronal toxicity was examined 24 h post-injection. The whole brains were sectioned and carefully screened for toxicity by gallocyanine staining, even though toxicity was detected only at the expected site in the CA2 region of the hippocampus. No toxicity was observed in mouse brains injected with buffer alone or PrP monomers (Figure 5C, 5E, 5G, and 5I). PrP oligomers were highly toxic in both PrP-expressing and non-expressing mice, leading to an almost complete destruction of the pyramidal layer of neurons in the hippocampal region underneath the injection site (Figure 5D and 5H). Murine PrP oligomers were as toxic as ovine PrP oligomers (not shown). In vivo, PrP fibrils were also toxic but to a markedly milder extent than PrP oligomers (Figure 5B and 5F). To see if the neurons were dying by apoptosis, the cells were labeled with ApopTag BrdU that binds to DNA breaks, a hallmark of apoptosis. As seen in Figure 6J and 6K, hippocampal neurons exposed to the toxic PrP oligomers exhibited intense BrdU labeling, which indicates that the neurons underwent apoptosis. Furthermore, the levels of BrdU labeling also provided a direct comparison of the level of toxicity of PrP oligomers versus PrP fibrils, showing again that the oligomers were more toxic than the fibrils.

**Table 1:**
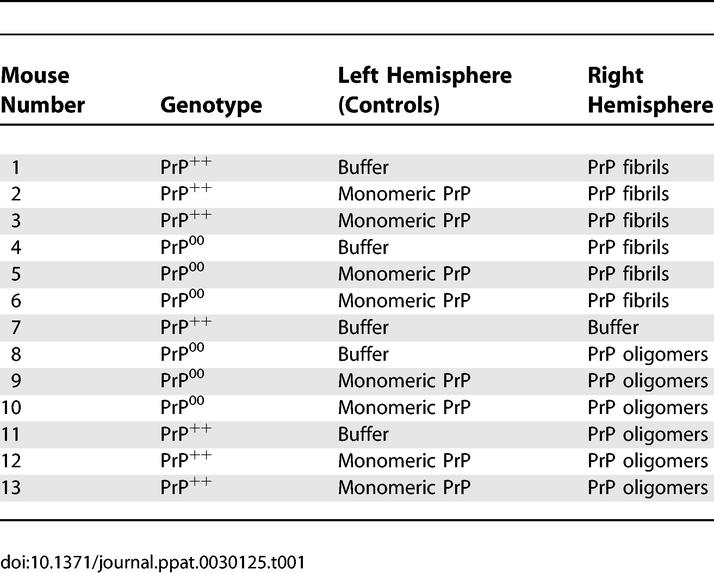
Experimental Set-Up of In Vivo Toxicity Assays

In summary, these data show in vivo that soluble particles of PrP oligomers exert a high intrinsic neurotoxicity, whereas large PrP fibrils exhibit low neurotoxicity, both independently of endogenous PrPc expression.

## Discussion

There is a growing belief that intermediates in the formation of the protease-resistant prion protein PrPsc (sometimes referred to as PrP* [25]), rather than PrPsc itself, are the pathogenic forms of PrP [10,11,13,14]. Moreover, there is evidence from other brain amyloidoses that soluble oligomeric forms of the disease-associated protein constitute the neurodegenerative trigger [21–24].

We tested this hypothesis using oligomeric ß-sheeted PrP preparations. Throughout the study, we used two different types of PrP preparations in order to obviate a possible bias due to the method of oligomer preparation. One was a murine tandem PrP construct that spontaneously formed oligomers, while the other was an ovine PrP, the oligomerisation of which was induced by thermal refolding into ß-sheeted PrP. Both have been characterized and used in previous studies [36,39]. We observed remarkably similar results with both types of oligomers in all the different experiments performed throughout this study. These oligomers were soluble in physiological buffers, showed mild PK resistance, and aggregated into insoluble amyloid fibrils upon aging, therefore resembling presumed PrPc to PrPsc conversion intermediates. We first showed that ß-PrP oligomers, but not α-PrP monomers, are toxic to cortical neurons in culture, in accordance with previous studies [32,33]. They were approximately 30 times more toxic than PrP peptides (Figure 2E and [18]). We did not observe a species-specific effect of murine versus ovine oligomers, suggesting that the main toxicity mechanism does not depend on a homologous interaction between PrPc and the PrP oligomers. In fact, the toxicity was completely independent of endogenous PrP expression by the neurons (Figure 2). The toxic effect of the ß-PrP oligomers in PrP^+/+^ and PrP^0/0^ neurons could be reversed by blocking the 106–126 hydrophobic stretch of PrP with the Pri303 antibody, suggesting a direct role of this region in the toxicity. We have previously demonstrated the higher accessibility of hydrophobic clusters at the surface of our ß-PrP oligomers by 1-anilino 8-naphthalene sulfonic acid (ANS) fluorescence probing [36]. It is a phenomenon common to protein misfolding [40], and other authors have shown that the hydrophobic stretch 90–120 of PrP is available for antibody binding in ß-oligomers [28]. Moreover, we have data (not shown) suggesting that ß-PrP oligomers undergo an increased cellular uptake. One possible explanation is that the hydrophobic surface of the PrP oligomers favors their insertion into the lipid bilayer. This finding is in accordance with studies using PrP peptides encompassing the hydrophobic PrP domain, showing that they insert metastably into membranes and are toxic independently of PrPc expression [18,19]. Interestingly, the SAF84 antibody actually increased the toxicity of the PrP oligomers. This effect is not linked to its binding to cell surface PrPc, since it was observed in PrP-expressing and non-expressing cells. Because SAF84 binds to the S2-H2 hinge loop involved in the oligomerization process [41], a possible hypothesis is that it facilitates PrP oligomerisation and hence increases the toxicity.

Upon aging, toxic ß-PrP oligomers assembled into insoluble fibrils that were not toxic on our primary cultures of cortical neurons. This finding is in accordance with the fact that the soluble, non-fibrillar amidated version of the hydrophobic PrP 106–126 peptide is toxic [42] and with the emerging view that the pathogenesis of amyloidotic diseases is related to soluble oligomeric species rather than to high molecular weight protein assemblies [21,24]. We then wanted to verify in vivo the relevance of our in vitro findings. We performed stereotaxical injections of either the control solution, PrP monomers, PrP oligomers, or PrP fibrils in the supra-hippocampal region of C57BL/6 PrP^+/+^ or C57BL/6 PrP^0/0^ mice (Figure 5). First, we confirmed that ß-PrP oligomers are highly toxic in vivo, both in PrP expressing or non-expressing mice. Like the in vitro experiments, the vehicle solution and α-PrP monomers were not toxic to the neurons in vivo. As a comparison, 10 μg of the 118–135 PrP peptide was toxic in vivo to retinal neurons after intravitreal inoculation [43]. Second, we found that PrP fibrils were toxic in vivo, but clearly less toxic than ß-PrP oligomers. The same phenomenon was observed in PrP^+/+^ and PrP^0/0^ mice. This is clearly shown in Figure 5 by the differences in the extent of neuronal damage in the hippocampal cell layer and by the difference in BrdU labeling for apoptosis. The fact that PrP fibrils revealed some toxicity in vivo but not in vitro may be due to a different level of sensitivity of the neuronal subpopulation examined in either case (hipocampal versus cortical neurons), as well as an amplification of toxicity in vivo due to the presence of glial cells in the brain. Another explanation may be that, in vivo, the fibrils were partially broken down into smaller, more toxic aggregates.

Another study showed in vitro that the toxicity of PrP oligomers and fibrils was dependent on the expression of PrP [33], suggesting the existence of alternative pathways of toxicity. Interestingly, the PrPc dependency of the PrP-induced toxicity has always been a matter of debate [17,44–46]. However, because these authors also observed a different toxicity behavior of their PrP fibrils in vitro than we did, we reason that all these differences may be due to the fibrils corresponding to different variants of supramolecular PrP assemblies. These variants would expose differently their reactive interface and thus react differently with the cell surface. Different shapes of PrP fibrils might be related with the “prion strain” phenomenon, causing the brain to degenerate more or less rapidly and triggering the death of different subset of neurons. Hence, apparent differences between our in vitro study and that by Novitskaya et al. teaches us that we may be revealing only different components of the very complex phenomenon of neurodegeneration induced by amyloidotic proteins. Therefore, while the dissection of mechanisms in vitro is obviously important, in vivo approaches constitute the only way to assess their overall effect on the brain.

In vivo, endogenous PrP is required to generate PrP oligomers, but, as shown by our experiment where we have externally provided the toxic PrP species to PrP^0/0^ cells, not to induce neuronal death. This is also in accordance with findings in a transgenic mouse model where prion replication and death of PrP^0/0^ neurons occurred by exclusive astrocytic prion release [46]. In another study, conditional suppression of PrPc expression in neurons during murine prion infection led to a halt in the neurodegenerative process and behavioral alterations [47,48]. In this model however, even if PrPsc continued to accumulate, it was found restricted to astrocytes, and the absence of supply of toxic PrP species in the vicinity of neurons was probably key to the neuroprotective effect.

Our in vivo findings are in accordance with the emerging consensus that during prion diseases, small undetectable PrP aggregates, rather than plaque-type PrP deposits, are responsible for neuronal dysfunction and death. This is supported by the lack of correlation between neuronal death and the observation of PrP plaques in vivo [9,49,50]. Moreover, highly aggregated extracellular deposits of PrP in scrapie-infected “anchorless” transgenic mice exhibit very low toxicity, if any at all [51]. Even if not toxic, this amyloid PrP or another yet unknown component is infectious, as evidenced by transmission to wild-type mice. Interestingly, another recent study suggests dissociation also beteween the presence of Prp amyloid and prion infectivity [52]. In a previous study where focal PrPsc aggregates where found in PrP^0/0^ mice grafted with PrP^+/+^ tissue, no toxicity was observed, possibly due to the aggregation state of the PrPsc detected by immunohistochemistry [45].

The finding that soluble PrP oligomers, preceding the formation of PrP fibrils, are the main neurotoxic species in vivo, assigns prion diseases to the group of other brain amyloidoses, like Alzheimer and Parkinson disease, with regard to their mechanism of neurodegeneration [7]. The commonality of this mechanism is remarkable. It involves a conformational change of the protein monomer, leading to the formation of soluble aggregates, which become insoluble as the protofilaments grow into amyloid fibrils. An antibody recognizing common structural elements from different cytotoxic oligomers was able to inhibit their cellular toxicity, hinting at a commonality also in the primary targets of toxicity [22]. Because some amyloid proteins, like Aß, are located in the extracellular space, whereas others (α-synuclein) are cytosolic, it is likely that cell membranes that are accessible from both compartments constitute one of these targets. The early prefibrilar aggregates of HypF-N (a disease-unrelated protein used as a model to study aggregate-forming, pathogenic proteins) were shown to be able to permeate synthetic phospholipid membranes [53]. Recently, the physical mechanism of toxicity associated with the intermediate-size Aß peptide oligomers was found to be the formation of conducting pores in lipid bilayers [54].

Figure 6 reviews the most probable scenarios of PrP-induced toxicity, showing how our data feed into the context of earlier findings. In this model, the toxicity of the PrP oligomers would be 3-fold : the first scenario is linked to the conformational change of oligomeric PrP, resulting in the loss of the N-terminal flexibility in the oligomers, which thereby mimic the effect of ΔPrP constructs described in earlier studies (Figure 6, #3 and #4); the second is the membrane insertion and destabilization of PrP oligomers, similar to the effects of PrP peptides (Figure 6, #5); and the third relates to the intracellular effects of PrP oligomers, comparable to those described with cytoplasmic PrP (mainly Figure 6, # 6).

In normal cells, PrPc is thought to convey a survival message by interacting with a cell surface ligand, LPrP. Several studies have shown that the PrPc binding domain is located in the structured core of the protein while the activating domain is in the flexible N-terminus of the protein [20,55–57] (Figure 6, #1). One of the scenarios proposed is that in PrP^0/0^ mice, a hypothetical functional homologue of PrP (π) binds to LPrP and transduces the signal (Figure 6, #2). However, PrP^0/0^ mice engineered to express an N-terminally truncated PrP, or a PrP truncated in the most C-terminal part of the flexible domain (ΔPrP) or Doppel (a protein of the PrP supergene family that lacks the N-terminus equivalent of PrP), harbor a neurotoxic phenotype, because Doppel and ΔPrP bind to LPrP with higher affinity than π but lack the domain responsible for the transduction of the survival message (this is the so-called Shmerling or Doppel effect; Figure 6, #3). In PrP oligomers, the N-terminus of PrP is thought to be buried [36] and hence the oligomers would behave similarly to Doppel or ΔPrP with regard to their interaction with LPrP (Figure 6, #4). Cellular uptake of PrP oligomers is likely to induce intracellular toxicity by the accumulation of protein oligomers at the mitochondrial membrane, resulting in the release of cytochrome C and subsequent activation of the apoptotic cascade (Figure 6, #7), as suggested for Parkinson and Alzheimer diseases [58]. Finally, it has been shown that a highly concentrated intracellular abnormal PrP species is likely to end up accumulating in the aggresome/proteasome system [59,60]. Increased uptake and intracellular accumulation of PrP oligomers is likely to saturate intracellular degradation pathways like the proteasome, thereby triggering the apoptotic cascade (Figure 6, #6). The latter neurotoxic mechanism would also explain why most amyloid diseases are associated with old age, when there is likely to be an increased tendency of proteasome dysfunction and for proteins to become misfolded or damaged, in conjunction with the reduced efficiency of the molecular chaperone and unfolded protein responses [61,62].

The present study establishes ß-PrP oligomers as a major neurotoxic species in vitro and in vivo, which likely represents the culprit PrP* responsible for the development of transmissible spongiform encephalopathy–linked neurodegeneration. Targeting ß-PrP oligomers, and their hydrophobic domain in particular, will allow researchers to devise rational neuroprotective treatments for these highly debilitating diseases.

## Materials and Methods

### Protein preparations.

The recombinant proteins used in this study were a monomeric mouse PrP sequence (23–231), a mouse tandem PrP composed of two monomeric sequences linked head to tail from the carboxy terminal to the amino terminal by a linker (flexible sequence) to allow for proper folding of the dimer, an α-helical sheep PrP, and a ß-sheeted version of the sheep PrP. The grb-2 protein and the linker sequence flanked at both sides by ten amino acids of the PrP sequence were used as control proteins.

Tandem PrP consists of two covalently linked murine PrP sequences without N- and C-terminal signal peptides. This recombinant protein was expressed and purified as previously described [41]. The proteins were stored at −20 °C in their elution buffer (8 M urea, 20 mM sodium phosphate, 500 mM sodium chloride, 500 mM imidazole, [pH 6.3]) until needed (see below).

The ovine PrP full-length protein was purified as described previously [64]. Briefly, the gene encoding the full-length ARQ variant (A136 R154 Q171) was cloned in pET 22b+ and expressed by IPTG induction in the *Escherichia coli* BL21 DE3 strain. After lysis, sonication, and solubilization of the inclusion bodies with urea, purification and renaturation of the prion protein were performed on an Ni Sepharose column by heterogeneous phase renaturation, taking advantage of the intrinsic affinity of the full-length protein for Ni.

For conversion, PrP (pH 7.2 in 20 mM MOPS) was heated at 72 °C for 15 min and cooled to room temperature. Fourier transform infrared spectra confirmed that in these conditions, PrP forms oligomeric ß-sheeted PrP. The characterization and mechanism of formation of these ß-sheeted oligomers is published elsewhere [36]. Briefly, they form discrete 12-mer and 36-mer species, are oblate-shaped, have distinct secondary structure features, and display exposures of hydrophobic clusters.

The synthetic peptides used in this study were mouse PrP 105–132: KTNLKHVAGAAAAG-AVVGGLGGYMLGSA and mouse PrP scrambled 105–132: NGAGKAGMVGLYGAHG-ATAKVSLVGALA. They were prepared as described in [18].

### Storage and dialysis of proteins.

Mouse proteins were stored at −20 °C in their elution buffer (see above). The proteins were dialyzed just before use against ultrapure water or 10 mM sodium acetate (pH 4.5), and the concentration was obtained by the micro BCA protein assay. The sodium acetate did not affect the pH of the culture medium or the viability of the neurons at the dilutions used.

Sheep PrPs were produced just before use and kept at 4 °C for a maximum of 30 d.

### Primary mouse cortical cultures.

PrP^+/+^ mice used in this study were wild-type C57BL/6 mice. PrP^0/0^ mice were obtained by backcrossing PrP knockout mice, kindly provided by Charles Weissmann, with C57BL/6 mice over nine generations, and are therefore named C57BL/6 PrP^0/0^ mice. Primary cortical cells were extracted from 15-d-old mouse embryos. Cortices were dissected under a binocular microscope in Ca^2+^/Mg^2+^-free PBS (Invitrogen, http://www.invitrogen.com/) supplemented with glucose at a final concentration of 3%. Then, they were carefully freed of meninges and incubated in trypsin/EDTA solution (Eurobio, http://www.eurobio.fr/) for 10 min at 37 °C. The trypsin was removed and the leftover was inactivated with DMEM (Dulbecco's Modified Eagle's Medium, Invitrogen) containing 4.5 g/l glucose, Glutamax-I, and 1% FCS (fetal calf serum, Invitrogen). Cells were then mechanically dissociated using a flame-narrowed Pasteur pipette in the same culture medium. The cell suspension was then centrifuged and the pellet resuspended in DMEM supplemented with B27 (Invitrogen) and 3% FCS. Cells were seeded on plates coated with 10 μg/ml of poly-D-lysine (Sigma, http://www.sigmaaldrich.com/) initially in DMEM supplemented with B27 (2%), FCS (3%), and 100 U/ml penicillin/streptomycin. After 2 d, the culture medium was replaced by serum-free DMEM containing N2 supplements (1%; Invitrogen) and penicillin/streptomycin. Cultures were kept in a 37 °C water-saturated incubator at 5% CO_2_.

### In vivo neurotoxicity experiments.

Female C57BL/6 PrP^+/+^ and C57BL/6 PrP^0/0^ mice (see paragraph above) were anesthetized with isoflurane (1%–2.5%) and positioned on a stereotaxic frame. Once the bregma was identified and holes drilled, 2 μl of 1 mg/ml of various PrP preparations or the same volume of buffer were injected into the hippocampus of the ipsi and controlateral hemispheres (1.5 mm posterior, +/−2.00 mm lateral, and 1.75 mm ventral to bregma, Figure 5A) at a rate of 0.4 μl/min. The animals were killed by cervical dislocation at 24 h post-injection. The brains were dissected out, fixed in 4% buffered paraformaldehyde, and paraffin embedded. For the visualization of the neurons, 5-μm horizontal sections from the mid-brain region were stained for nucleic acids with gallocyanine according to standard protocols. To test for apoptosis, adjacent sections of selected brain sections were analyzed with the ApopTag BrdU kit according to the manufacturer's instructions (Molecular Probes, http://probes.invitrogen.com/). Slides were examined on an epifluorescence Zeiss microscope (http://www.zeiss.com/). All animal experiments were performed in accordance with national and European Union (EU) regulations.

### Proteinase sensitivity of PrP species.

Analysis of PK resistance was performed by incubating 10 μg/ml of each protein for 15 min in the presence of various concentrations of PK at 37 °C. The samples were then precipitated with four volumes of methanol, resuspended in the loading buffer, and analyzed by western blot with the monoclonal antibodies 8G8 and Pri917 for the ovine and mouse PrP samples, respectively.

### In vitro neurotoxicity experiments.

For toxicity monitoring, cells were seeded at a density of 7 × 10^4^ cells per wells in a 96-well poly-D-lysine–coated plate (in each plate, only the 60 wells in the center contained cells and the outer wells were filled with PBS to prevent any drying). After 5 d in culture, neurons were incubated with the different recombinant PrP proteins for 72 h. In order to keep steady culture medium concentrations, the proteins were diluted in 2× culture medium and the proper volume of water and vehicle solution were added to get a final concentration of 1×. For controls, the cells were left untreated or were exposed to an equivalent volume of vehicle solution.

### Cell survival assays.

After exposure of the neurons to the proteins, the viability was measured either with MTT or with WST-1. For MTT, the medium was replaced with 500 μg/ml of 3,[4,5 dimethylthiazol-2*yl*]-2,5 diphenyltetrazolium bromide (MTT; Sigma) dissolved in PBS. After 2 h of incubation at 37 °C, the solution was removed and the blue formazan was solubilized with an isopropanol/HCL 1N (92:8) solution. Then, the optical density was measured at 540 nm with a reference wavelength of 630 nm. For WST-1 (Roche, http://www.roche.com/), 10 μl of the reagent was added directly to the culture medium containing the cells and incubated for 1.5 h. Then, optical density measures were taken at 450 nm against a reference wavelength of 630 nm. In this case, a negative control, consisting of DMEM without cells, was used and subtracted from all samples.

### Visualization of apoptotic cells.

This was performed by staining cell nuclei with Hoechst 33342. Cells were seeded in poly-D-lysine-coated 8-well Labtek (Labtek II; Nalgene Nunc International, http://www.nalgenunc.com/) culture dishes at a density of 1.6 × 10^5^ cells/wells. After treatment with the recombinant proteins, the cells were mounted with Vectashield (Vector Laboratories, http://www.vectorlabs.com/) supplemented with 5 μg/ml of Hoechst 33342 reagent (Molecular Probes). The slides were visualized using an axiovert (Zeiss) fluorescence microscope.

### Rescue experiments.

All monoclonal PrP antibodies used in this study have been purified with Protein-A or Protein-G affinity columns and dialyzed against ultra-pure water. These steps were carried out to remove growth factors and anti-microbial agents from the antibody solutions. Antibodies were added directly to the culture media containing the recombinant proteins of interest. Then these were incubated with the cells for 72 h.

### Electron microscopy of PrP samples.

All protein samples were first added (0.2–1.5 mg/ml) to serum-free DMEM containing N2 supplements (to be in the same conditions as the neurotoxicity experiments) and incubated for 2 h at 37 °C. Following this step, a 10-μl aliquot of protein preparation was applied to formar- and carbon-coated grids. The excess fluid was drained with filter paper and the sample was stained for 1 min with 2% uranyl acetate. The grid was air-dried and examined in a Philips EM CM120 at 80 kV at a magnification of 15–75000.

### Solubility assay of PrP samples.

Young and aged PrP dimers were centrifuged at 100,000*g* for 1 h and the supernatant was separated from the pellet. The PrP was then visualized by western blot with the antibody 4H11.

### Thioflavine T fluorescence measurements.

Thioflavine T fluorescence mesurements were performed at 20 °C on a Jasco 6200 spectrofluorimeter (http://www.jascoint.co.jp/) with a 1 mm × 10 mm optical path-length cuvette. The concentation of protein was adjusted to 15 μM (equivalent monomer) for each species before incubation with 15 μM thioflavine T. Excitation was performed at 432 nm.

## Abbreviations

LPrP: PrP ligand
PK: proteinase K
PrP: prion protein
PrPsc: disease-associated form of PrP
